# Reduced anticoagulation strategy is associated with a lower incidence of intracerebral hemorrhage in COVID-19 patients on extracorporeal membrane oxygenation

**DOI:** 10.1186/s40635-023-00525-3

**Published:** 2023-06-12

**Authors:** Daniel A. Hofmaenner, David Furfaro, Lennart C. Wild, Pedro David Wendel-Garcia, Elias Baedorf Kassis, Ameeka Pannu, Tobias Welte, Rolf Erlebach, Klaus Stahl, Edward Wilson Grandin, Christian Putensen, Reto A. Schuepbach, Shahzad Shaefi, Sascha David, Benjamin Seeliger, Christian Bode, for the BonHanZA study group

**Affiliations:** 1 Institute of Intensive Care Medicine, University Hospital Zurich, Raemistrasse 100, 8091 Zurich, Switzerland; 2 Division of Pulmonary, Critical Care, and Sleep Medicine, Department of Medicine, Beth Israel Deaconess Medical Center, Harvard Medical School, Boston, MA USA; 3 Department of Anesthesia and Intensive Care Medicine, University Hospital Bonn, Bonn, Germany; 4 Department of Anesthesia, Critical Care and Pain Medicine, Beth Israel Deaconess Medical Center, Harvard Medical School, Boston, MA USA; 5 Department of Respiratory Medicine, Hannover Medical School and Member of the German Centre for Lung Research, Biomedical Research in End-Stage and Obstructive Lung Disease Hannover (BREATH), Hannover, Germany; 6 Department of Gastroenterology, Hepatology and Endocrinology, Hannover Medical School, Hannover, Germany; 7 Division of Cardiovascular Medicine, Beth Israel Deaconess Medical Center, Harvard Medical School, Boston, USA

**Keywords:** Acute respiratory distress syndrome, Extracorporeal membrane oxygenation, COVID-19, Anticoagulants, Hemorrhage, Bleeding, Endothelium, Vascular

## Abstract

**Background:**

Optimal anticoagulation strategies for COVID-19 patients with the acute respiratory distress syndrome (ARDS) on venovenous extracorporeal membrane oxygenation (VV ECMO) remain uncertain. A higher incidence of intracerebral hemorrhage (ICH) during VV ECMO support compared to non-COVID-19 viral ARDS patients has been reported, with increased bleeding rates in COVID-19 attributed to both intensified anticoagulation and a disease-specific endotheliopathy. We hypothesized that lower intensity of anticoagulation during VV ECMO would be associated with a lower risk of ICH. In a retrospective, multicenter study from three academic tertiary intensive care units, we included patients with confirmed COVID-19 ARDS requiring VV ECMO support from March 2020 to January 2022. Patients were grouped by anticoagulation exposure into higher intensity, targeting anti-factor Xa activity (anti-Xa) of 0.3–0.4 U/mL, versus lower intensity, targeting anti-Xa 0.15–0.3 U/mL, cohorts. Mean daily doses of unfractionated heparin (UFH) per kg bodyweight and effectively measured daily anti-factor Xa activities were compared between the groups over the first 7 days on ECMO support. The primary outcome was the rate of ICH during VV ECMO support.

**Results:**

141 critically ill COVID-19 patients were included in the study. Patients with lower anticoagulation targets had consistently lower anti-Xa activity values over the first 7 ECMO days (*p* < 0.001). ICH incidence was lower in patients in the lower anti-Xa group: 4 (8%) vs 32 (34%) events. Accounting for death as a competing event, the adjusted subhazard ratio for the occurrence of ICH was 0.295 (97.5% CI 0.1–0.9, *p* = 0.044) for the lower anti-Xa compared to the higher anti-Xa group. 90-day ICU survival was higher in patients in the lower anti-Xa group, and ICH was the strongest risk factor associated with mortality (odds ratio [OR] 6.8 [CI 2.1–22.1], *p* = 0.001).

**Conclusions:**

For COVID-19 patients on VV ECMO support anticoagulated with heparin, a lower anticoagulation target was associated with a significant reduction in ICH incidence and increased survival.

**Supplementary Information:**

The online version contains supplementary material available at 10.1186/s40635-023-00525-3.

## Background

Despite an impressive gain of knowledge throughout the COVID-19 pandemic, the management of COVID-19 patients with acute respiratory distress syndrome (ARDS) remains challenging [1]. For these patients, venovenous extracorporeal membrane oxygenation (VV ECMO) can be a potential life-saving intervention facilitating lung protective ventilation [2, 3]. However, the use of ECMO is associated with serious complications such as an increased bleeding risk and hemotrauma [4]. In addition, patients with critical COVID-19 exhibit unique abnormalities in coagulation which can increase the risk of both bleeding and thrombotic events [5, 6], thereby challenging clinicians when VV ECMO is initiated for patients with COVID-19 ARDS [7].

During the early phase of the pandemic the pro-thrombotic features of COVID-19 were among the key characteristics that clinically separated these patients from other viral ARDS patients [8]. Intensified anticoagulation strategies in COVID-19 patients, including those requiring VV ECMO support became a common practice despite a lack of clear evidence [9].

Recent studies have highlighted an increased incidence of intracerebral hemorrhage (ICH) in COVID-19 patients (20%) compared to individuals with other viral pneumonia (6%) on VV ECMO support [10, 11], demonstrating ICH rates significantly above pre-pandemic ECMO trials [12]. Since we observed devastating mortality rates of up to 90% in patients with ICH [10], our ECMO centers empirically lowered the anticoagulation target ranges for COVID-19 patients on ECMO support from anti-factor Xa activity (anti-Xa) of 0.3–0.4 U/mL to anti-Xa activity of 0.15–0.3 U/mL. We hypothesized that this switch towards a less intense anticoagulation strategy was associated with a lower occurrence rate of ICH.

## Methods

### Study design and study subjects

This retrospective, multicenter study was conducted at the intensive care units (ICU) of the University Hospital Zurich (Switzerland), University Hospital Bonn (Germany) and Beth Israel Deaconess Medical Center (BIDMC) in Boston, MA (United States of America). The study was performed and data were acquired with approval of all responsible local ethics committees (Kantonale Ethikkommission Zürich, BASEC 2021-00825; Ethikkommission University Hospital Bonn number 196/21; BIDMC Committee on Clinical Investigation under IRB protocol 2017P000310) and shared through an executed Data Use Agreement.

Adult patients (> 18 years) with COVID-19 ARDS requiring VV ECMO support were assessed for eligibility between March 2020 and January 2022, independent of the chosen anticoagulation strategy. Patients were excluded if they required venoarterial ECMO or had non-heparin anticoagulation. COVID-19 was determined by real-time reverse transcriptase-polymerase chain reaction (RT-PCR) positivity of nasopharyngeal and/or pharyngeal swabs, tracheobronchial secretions or bronchoalveolar lavage. When ICH or other cranial pathology was clinically suspected, cranial computed tomography (CCT) was immediately performed. No routine surveillance cranial imaging was performed at any center.

### Baseline data collection

Using the in-hospital patient data management systems, clinical data were gathered including demographics, elements of the Sequential Organ Failure Assessment (SOFA) score [13] at VV ECMO initiation, respiratory parameters, laboratory values, the need for vasopressors, anti-inflammatory treatment (dexamethasone and tocilizumab use) and the need for renal replacement therapy at VV ECMO initiation.

### Anticoagulation strategies

All centers utilized standard operating procedures for adjustment of anticoagulation on VV EMCO which prioritized anti-Xa activity targets and unfractionated heparin (UFH) as a primary anticoagulant in accordance with Extracorporeal Life Support Organization (ELSO) and society recommendations [14–16]. Antithrombin III and fibrinogen levels were monitored and repleted to target levels per individual institutional protocols. When patients showed signs of bleeding and had fibrinogen levels below 1–1.5 g/L, fibrinogen was substituted to target levels of 1.5–2 g/L. Antithrombin III was measured in case of suspected heparin resistance and was substituted if the antithrombin III activity was < 30%.

Patients were divided into two cohorts based on the anticoagulation intensity as defined by the anti-Xa target corridors: higher intensity 0.3–0.4 U/mL (high anti-Xa) versus lower intensity 0.15–0.3 U/mL (low anti-Xa). The higher intensity group consisted of patients managed earlier in the pandemic when there was an effort to counteract the profound procoagulant effects of COVID-19 with higher systemic anticoagulation [17, 18]. Lower intensity anti-Xa levels were targeted after the observation of high incidences of ICH during VV ECMO support compared to non-COVID-19 viral ARDS [10].

### Outcome parameters

The primary outcome was the incidence of ICH, which was defined as any hemorrhage identified on CT scanning of the brain during VV ECMO support. For both groups (high vs. low anti-Xa), we analyzed the occurrence rates of ICH (including major and minor bleedings), survival probability and predictors of survival. An ICH was defined as major if it fulfilled one of the following: (1) requiring neurosurgical intervention; (2) imaging was ordered due to clinical neurologic deficit; (3) imaging demonstrated a clinically relevant bleeding excluding microhemorrhage or minor subarachnoid hemorrhage without midline shifts; or (4) the bleeding was fatal and/or led to withdrawal of therapy [10]. All other ICH events not fulfilling these criteria were considered minor. Secondary outcomes included: (1) 90-day survival; (2) the cumulative and mean daily dose of UFH per kg bodyweight during the first 7 days of ECMO support, and (3) the median of effectively measured daily anti-Xa levels (as measured by the hospital laboratories), number of oxygenator changes and thromboembolic events.

### Statistical analysis

Data are presented as mean ± SD or median (25% to 75% IQR) depending on the distribution of data. Two-tailed *p* values of less than 0.05 were considered to represent statistical significance. Comparisons of population characteristics between the low and high anticoagulation target groups were performed using *t*-tests, Wilcoxon signed-rank tests and *χ*^2^ test, as appropriate. Comparison of the longitudinal course of effective anticoagulation by anti-Xa values over the first 7 ECMO days was approached by means of a linear mixed effects model [19]. Anti-Xa values were entered as the outcome variable and anticoagulation group and time was entered as independent fixed effects including the interaction between both. Per patient intercepts and center were included as a hierarchically nested random effect with patient nested within center. According to the raw data, a “steady state” of anti-Xa levels within the groups was reached after day 2, so natural cubic splines with a knot after day 2 were added to of the model using the “splines” R package. *P* values for individual fixed effects were obtained by Satterthwaite’s degrees of freedom method.

Risk factors and influence of anticoagulation on occurrence of ICH was analyzed by means of a competing risk regression model using ICH as the primary event and death without ICH as a competing event. As independent variables, anticoagulation group, dexamethasone use, tocilizumab use, SOFA score and female sex were entered with center as a cluster term [20].

90-day survival was compared between the higher- and lower-intensity anticoagulation groups using the Kaplan–Meier method and log-rank testing. For predicting ICU survival, a generalized linear mixed effect model was fitted using overall 90-day ICU mortality as the outcome variable and anticoagulation group, dexamethasone use, tocilizumab use, female sex, SOFA score, and occurrence of ICH (of any severity) as independent fixed effects. Per patient intercepts and center were included as a hierarchically nested random effect with patient nested within center.

Model fit was assessed using a likelihood ratio test (generalized linear model) or ANOVA test (linear mixed effect model) of the full model with the effects in question against a “null model”. Terms were retained only if they contributed to the model. Statistical analyses were performed using the R environment for statistical computing version 4.0.4 (R Foundation for Statistical Computing, Vienna, Austria).

## Results

In total, 141 critically ill COVID-19 patients receiving VV ECMO support were included in the study and underwent analysis (Table 1). There were 93 patients in the high anti-Xa group and 48 patients in the low anti-Xa group, respectively. The mean age at admission was 53.8 ± 11.5 years. One-third of patients were female and 74.5% of patients were on vasopressors. Mean SOFA score was 11.8 (± 3.38). Median length of ICU stay was 37 days (interquartile range [IQR] 33–57 days) and median ECMO runtime was 19 days (IQR 9–27 days). Patients in the low anti-Xa group were significantly younger, more often female, had slightly lower SOFA scores, and had lower levels of some inflammatory markers at ECMO initiation including C-reactive protein and ferritin (Table 1).

**Table 1:** Demographics, ICU characteristics/treatment, respiratory and laboratory parameters at ECMO initiation according to groups (high vs. low anti-Xa)

	High anti-Xa (*n* = 93)	Low anti-Xa (*n* = 48)	Total (*n* = 141)	*p* value
Demographics				
Age (y)	56.1 (10.95); range 19–77	49.6 (11.48); range 25–67	53.9 (11.50); range 19–77)	0.002
Female gender	19 (20.4%)	27 (56.2%)	46 (32.6%)	< 0.001
Body mass index (kg/m^2^)	31.9 (7.8)	31 (5.7)	31.6 (7.1)	0.451
Respiratory parameters at ECMO initiation				
Positive end-expiratory pressure (mbar)	14 (3.7)	14.1 (3.9)	14.1 (3.7)	0.913
Plateau pressure (mbar)	31 (4.3)	29.9 (4.8)	30.7 (4.5)	0.202
paCO_2_ (kPa)	66.7 (19.3)	68.7 (23.9)	67.4 (20.9)	0.602
pH	7.3 (0.1)	7.3 (0.3)	7.3 (0.2)	0.088
ICU characteristics/treatment				
Vasopressor need	72 (77.4%)	33 (68.8%)	105 (74.5%)	0.263
Dose of norepinephrine (mcg/min/kg)	0.17 (0.21)	0.12 (0.19)	0.16 (0.21)	0.213
Renal replacement therapy	19 (20.4%)	5 (10.4%)	24 (17.0%)	0.134
SOFA score	12.2 (3.5)	11.1 (3.1)	11.8 (3.4)	0.07
ICU length of stay (days)	33 (IQR 25–48)	41 (IQR 33–57)	37 (IQR 26–50)	0.044
ECMO runtime (days)	17 (IQR 17–29)	22 (IQR 14–32)	19 (IQR 9–27)	0.043
Dexamethasone treatment	64 (68.8%)	47 (97.9%)	111 (78.7%)	< 0.001
Tocilizumab treatment	2 (2.2%)	16 (33.3%)	18 (12.8%)	< 0.001
Laboratory parameters at ECMO initiation				
C-reactive protein (mg/L)	212.9 (123.5)	150.8 (136.3)	191.3 (131)	0.009
Ferritin (mcg/L)	1925.5 (1805.5)	1168.0 (1052.2)	1697.5 (1648.2)	0.032
Interleukin 6 (ng/L)	4737.3 (21,973.6)	6159.2 (13,175.1)	5249.8 (19,195.9)	0.744
Leukocytes (G/L)	14.4 (7.4)	13.9 (7.6)	14.3 (7.4)	0.719
Thrombocytes (G/L)	227.6 (111.5)	249.2 (124.7)	235 (116.2)	0.297
D-dimers (mg/L)	35.1 (239)	92 (544)	53.6 (365.5)	0.427
Lactate dehydrogenase (U/L)	544.9 (219.9)	529 (216.8)	539.3 (218.1)	0.701
Lactate (mmol/L)	2.2 (2.3)	2.0 (1.3)	2.1 (2)	0.609

Values are presented as *n* (%) for categorical data and mean (standard deviation) or median (interquartile range IQR) for continuous data

ECMO: extracorporeal membrane oxygenation; paCO_2_: partial pressure of arterial carbon dioxide; SOFA score: Sequential Organ Failure Assessment Score

**Fig. 1:**
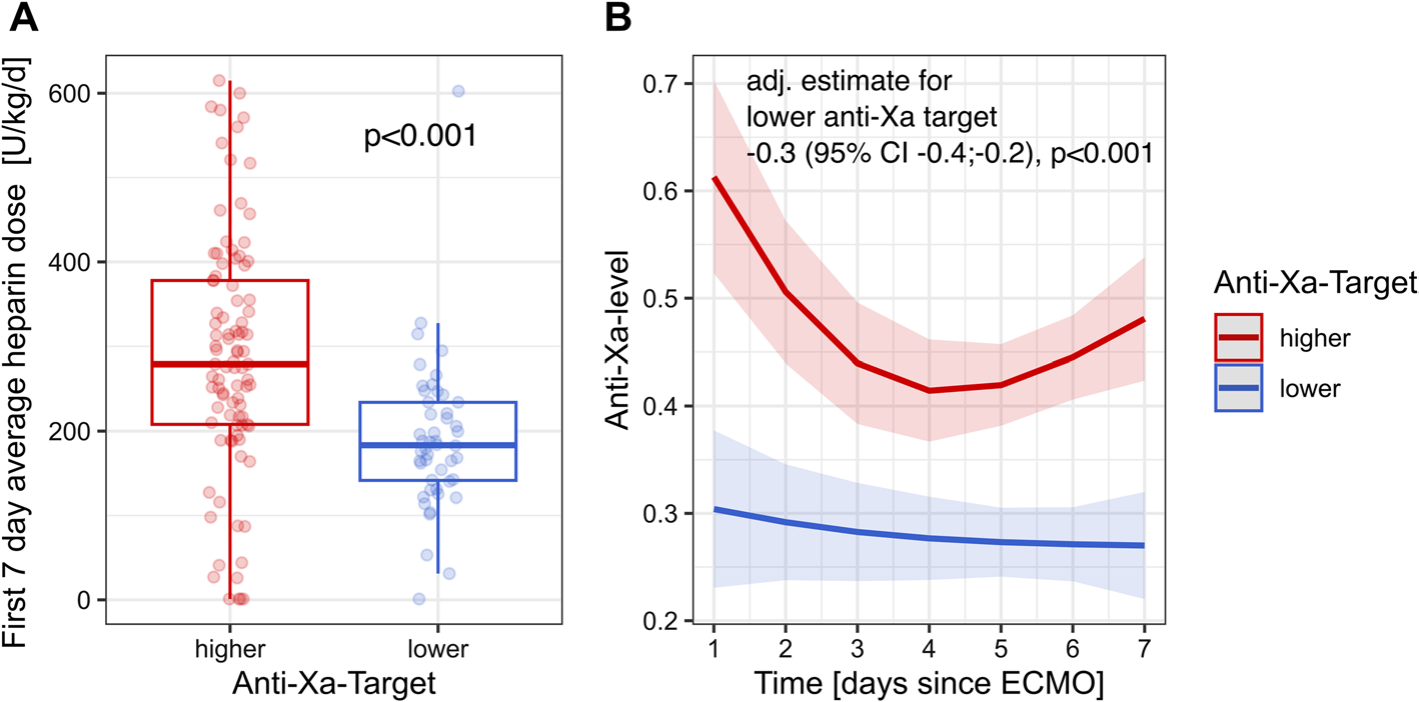
The cumulative mean daily dose of UFH per kg bodyweight during the first 7 days of ECMO support according to anticoagulation target group (**A**). Median of effectively measured daily anti-Xa activities according to anticoagulation target groups (**B**)

The cumulative mean daily dose of UFH per kg bodyweight during the first 7 days of ECMO support was significantly higher in patients of the high anti-Xa group compared to patients of the low anti-Xa group (Fig. 1A). Over the first 7 days of ECMO support, the median of effectively measured daily anti-Xa activities between the groups was within the respective target ranges, and thereby significantly higher in patients of the high anti-Xa group (adjusted estimate for lower target group − 0.3 [95% CI − 0.4; − 0.2], *p* < 0.0001) (Fig. 1B and Additional file 1: Table S1).

Overall, 18 patients suffered from a thromboembolic event on ECMO support. Thromboembolic complications and types of thromboembolic events during ECMO support were similar between the high- and low anti-Xa groups (Table 2). In the high anti-Xa group, 41.9% of the patients had at least one oxygenator change, while in the low anti-Xa group, 60.4% of the patients had at least one oxygenator change (0.74 vs. 1.25 oxygenator changes per ECMO run, *p* = 0.028). There were no reported adverse events associated with oxygenator changes.

**Table 2:** Thromboembolic complications and type of thromboembolic events during ECMO support according to anti-Xa groups (high vs. low anti-Xa)

	High anti-Xa (*n* = 93)	Low anti-Xa (*n* = 48)	Total (*n* = 141)
Thromboembolic events			
Any event overall	12 (12.9%)	6 (12.8%)	18 (12.9%)
Type of thromboembolic event	12 events	6 events	18 events
Pulmonary embolism	5 (41.7%)	1 (16.7%)	6 (33.3%)
DVT	5 (41.7%)	4 (66.7%)	9 (50%)
ECMO lines	1 (8.3%)	0 (0.0%)	1 (5.6%)
Arterial thrombus	1 (8.3%)	0 (0.0%)	1 (5.6%)
Cardiac thrombus	0 (0.0%)	1 (16.7%)	1 (5.6%)

Values are presented as *n* (%) for categorical data

*DVT* deep vein thrombosis, *ECMO* extracorporeal membrane oxygenation

*P*-value for thromboembolic events overall and for type of thromboembolic event 0.982 and 0.373, respectively (Groups compared by Pearson’s Chi-squared test)

**Fig. 2:**
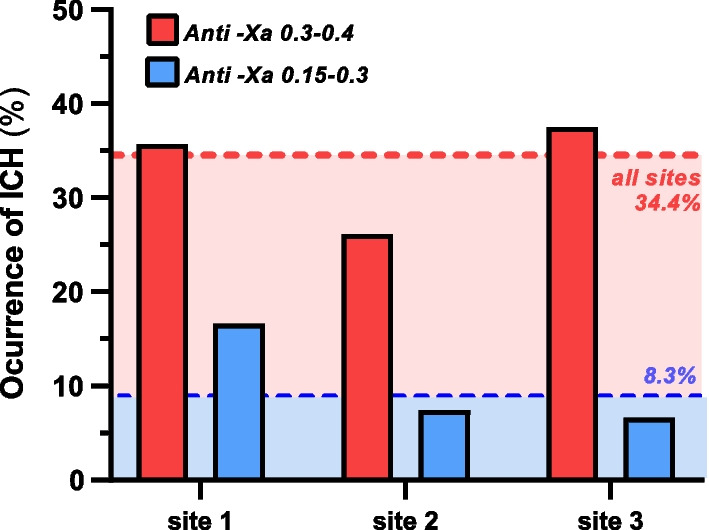
Rates of intracranial hemorrhages overall and for all study sites according to high or low anti-Xa. Site 1: University Hospital Bonn; Site 2: University Hospital Zurich; Site 3: Beth Israel Deaconess Medical Center Boston

ICH incidence was markedly decreased among patients with lower anticoagulation target ranges across all centers (Fig. 2). The overall incidence of ICH was 34% in the high anti-Xa group and dropped to 8% in the low anti-Xa group, with a decline of ICH rates at each site (Fig. 2).

**Fig. 3:**
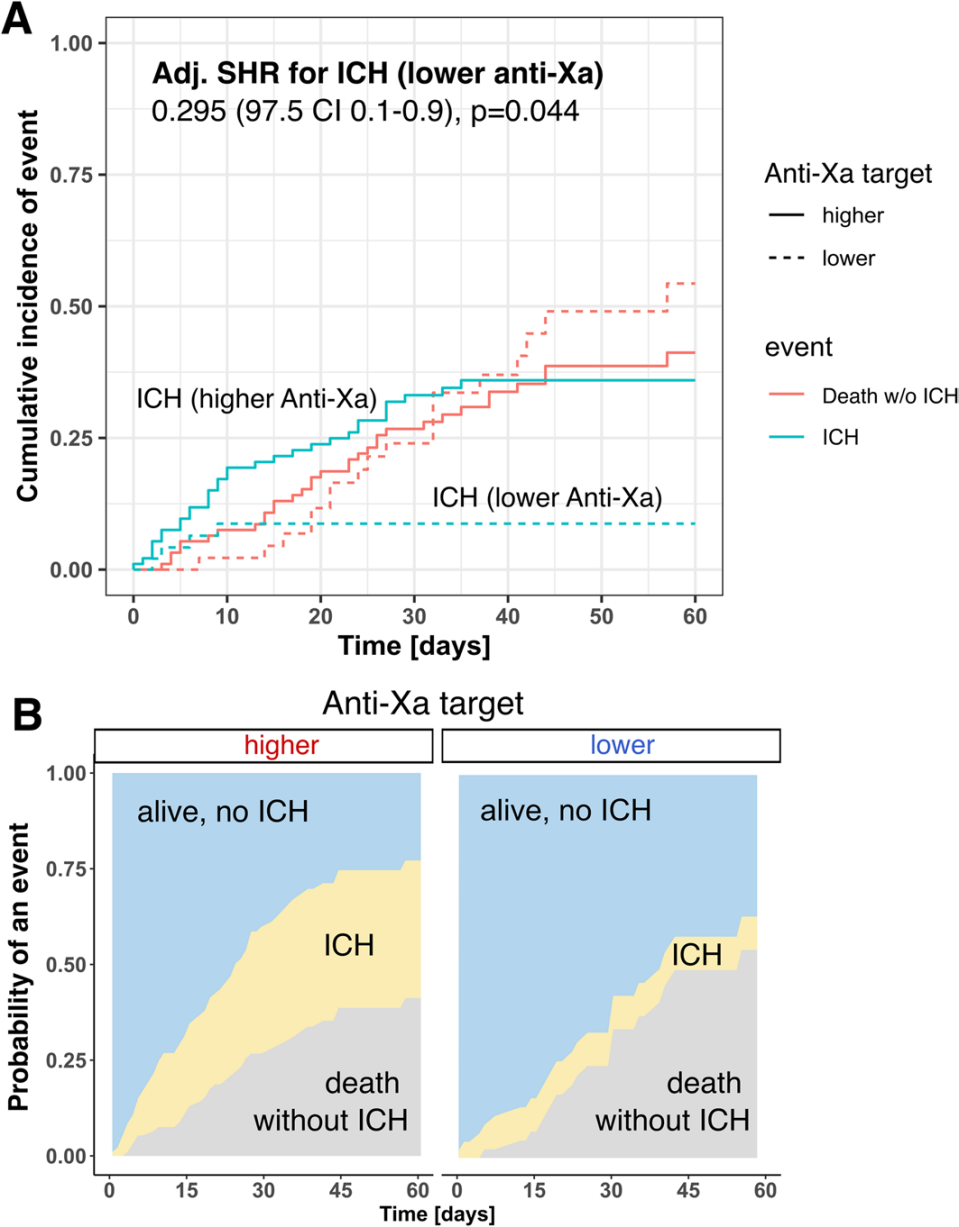
Competing risk regression model demonstrating incidences of intracranial hemorrhages (ICH) according to anticoagulation target group (**A**). Cumulative incidence of ICH and death without ICH) as multistate comparison is shown in **B**

In the competing risk regression model, the adjusted subhazard ratio for the occurrence of ICH was 0.295 (97.5% CI 0.1–0.9, *p* = 0.044) for patients in the low anti-Xa group, when treating death without ICH as a competing event (Fig. 3 and Additional file 2: Table S2). Median platelet counts at the time of ICH were normal and similar between the groups (110 (78–206) vs 126 (69–215) × 10^3^/µL, *p* = 0.934).

Overall, ICU mortality was 67.7% in the high anti-Xa group and 45.8% in the low anti-Xa group. Detailed mortality reasons for both anti-Xa groups are demonstrated in Table 3.

**Table 3:** Detailed mortality reasons according to anti-Xa groups (high vs. low anti-Xa)

	High anti-Xa (*n* = 93)	Low anti-Xa (*n* = 48)	Total (*n* = 141)
Mortality reason			
Fatal ICH	12 (12.9%)	0 (0%)	12 (8.5%)
Septic shock	23 (24.7%)	9 (18.8%)	32 (22.7%)
Refractory ARDS	21 (22.6%)	9 (18.8%)	30 (21.3%)
Other	7 (7.5%)	4 (8.3%)	11 (7.8%)

Values are presented as n (%) for categorical data

*ARDS* acute respiratory distress syndrome, *ICH* intracerebral hemorrhage

**Fig. 4:**
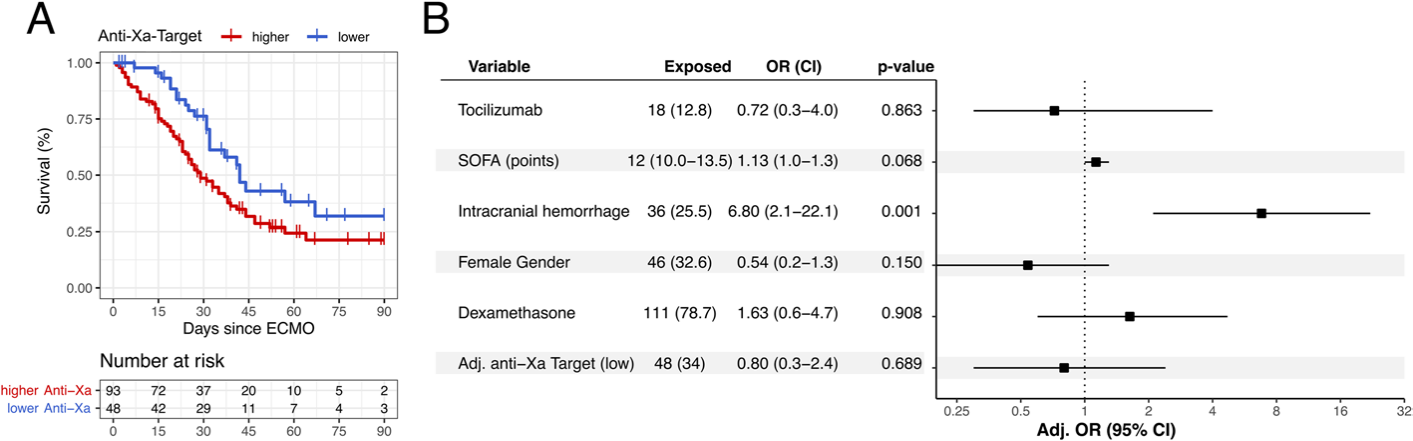
Generalized linear mixed effects model. Kaplan–Meier survival curves stratified by high and low anti-Xa targets demonstrating survival differences between the two anticoagulation groups (log-rank test, *p* = 0.022) (**A**). Occurrence of ICH was the main factor associated with mortality (adjusted odds ratio [OR] 6.8 [CI 2.1–22.1], *p* = 0.001) (**B**)

In the generalized linear mixed effects model, 90-day survival was significantly higher in the second observational period, where patients were subject to lower anticoagulation targets (log-rank test *p* = 0.022) (Fig. 4). Adjusted analysis of co-variables of the model revealed that the occurrence of ICH was the main factor associated with (odds ratio [OR] 6.8 [CI 2.1–22.1], *p* = 0.001) (Fig. 4).

## Discussion

In this retrospective multicenter analysis, we found that targeting a lower anti-Xa activity of 0.15–0.3 U/mL compared with a higher anti-Xa of 0.3–0.4 U/mL was associated with a lower incidence of ICH in COVID-19 ARDS patients receiving VV ECMO support. Furthermore, the clinical factor with the strongest association with inpatient mortality was the occurrence of ICH, and patients in the low anti-Xa group had a higher 90-day survival.

In light of the unique immune-thrombotic features of COVID-19, including severe endotheliopathy and coagulopathy [21], the hemostatic management of these patients remains challenging. When VV ECMO support is required for patients with COVID-19 ARDS, the need to prevent clotting of the extracorporeal circuit components increases complexity along with the fact that device-associated effects such as hemotrauma can increase bleeding and coagulation risk. High-dose heparinization in ARDS patients with VV ECMO support is associated with lower rates of oxygenator changes when compared to a low-dose heparinization strategy, but bears the risk of increased bleeding [22].

In the early phase of the COVID-19 pandemic, critically ill patients regularly underwent intensified therapeutic anticoagulation owing to the proposed procoagulant effects of the virus and an observed increase in fatal thromboembolic events [23]. However, several studies (including large platform trials) in critically ill COVID-19 patients subsequently highlighted increased rates of relevant bleeding complications, including ICH, without a detectable benefit with regard to the prevention of embolism [5, 10, 24, 25]. In our recent multicenter study [10], we reported a very high incidence (20%) of ICH in COVID-19 patients during VV ECMO support, with around 50% of the bleeding events being classified as major. Notably, 90% of ICH events, regardless of severity, were associated with mortality. Another recent study demonstrated an increased prevalence of bleeding events and ICH as causes of death in COVID-19 patients requiring ECMO support compared to those without ECMO support [26].

It has been speculated that this unacceptably high bleeding rate was driven by both intensified anticoagulation and the hosts’ vascular vulnerability as a characteristic feature of COVID-19 [27]. The latter notion is supported by the observation that in non-COVID-19 ARDS patients requiring ECMO support, a higher anticoagulation target was not associated with an increased ICH rate [26]. In parallel, large multicenter trials failed to demonstrate clear outcome benefits in critically ill COVID-19 treated with therapeutic anticoagulation compared to standard thromboprophylaxis [25].

In this current analysis, the lower anti-Xa target was achieved and the daily UFH use per kg body weight was reduced by a third between the high and low anti-Xa groups. This reduction in absolute heparin exposure was associated with a substantial reduction in the observed ICH rate from 34 to 8%. Our findings support previous observations that less aggressive anticoagulation strategies are safe and effective in critically ill COVID-19 patients [25]. The primary use of anti-Xa levels to monitor anticoagulation in patients with COVID-19 is consistent with international guidelines and is physiologically reasonable, since procoagulant factors and cytokines linked to COVID-19 ARDS may render aPTT values instable and may lead to overdosing of UFH [28]. Meta-analyses of anticoagulation strategies for ECMO suggest anti-Xa approaches may be associated with fewer bleeding complications without increased risk of thromboembolic events [29]. Indeed, we could not find any differences in thromboembolic complications between the high and low anti-Xa groups. This strengthened our rationale of targeting lower anti-Xa levels in COVID-19 patients on ECMO support without incurring more potentially harmful thromboembolic events. Our findings are in line with recent literature suggesting that bleeding events and not thromboembolic complications are primarily associated with mortality in critically ill COVID-19 patients on ECMO support [30]. There was a higher rate of oxygenator changes in the lower anti-Xa cohort, but there were no associated adverse events observed in this study. However, clinicians should take the likely higher risk of oxygenator changes into account and monitor potential oxygenator complications regularly. Future prospective research projects should continue to focus on the impact of lower anticoagulation targets on the occurrence of thromboembolic events or ECMO-associated complications such as device thrombosis or oxygenator changes [31].

Aside from anticoagulation targets, management strategies of critically ill COVID-19 patients have been adapted during the course of the pandemic. In order to counteract profound inflammation, dexamethasone and tocilizumab have been increasingly used, with varying effects on patient outcomes [32–34]. From a pathophysiological viewpoint, it would be plausible that attenuated inflammation could decrease the risk of ICH, independent of anti-Xa levels. Indeed, we found decreased CRP and ferritin levels in the lower anti-Xa group, probably reflecting a more frequent use of anti-inflammatory drugs later in the pandemic. Interleukin 6 (IL-6) levels were slightly higher in the low anti-Xa group, in line with observations that IL-6 serum levels even can increase after administration of tocilizumab [35]. Taking dexamethasone and tocilizumab use into account in our analysis, the occurrence of ICH was still the main risk factor for mortality, whereas use of dexamethasone and tocilizumab was not associated with better patient survival. However, these results should be interpreted with caution, as we cannot rule out residual confounders affecting treatment decisions and inflammation. Of note, ECMO runtime was slightly longer and organ support tended to decrease in the low anti-Xa group, both probably affecting ICH risk. Thus, further large-scale studies specifically including patients requiring ECMO support should clarify effects of different anticoagulation strategies and the use of anti-inflammatory medication on global patient outcomes such as ICU length of stay, the need for mechanical ventilation, long-term neurologic sequelae of patients affected by bleeding, and mortality [31].

Our study has to account for limitations. We had a relatively small population of COVID-19 patients of VV ECMO, but overall mortality was similar to previous VV ECMO studies [10, 12, 36] and the consistency of the results across multiple centers adds generalizability to our findings. The retrospective nature of this study prevented us from inferring causality, and we cannot exclude the possibility of unmeasured confounders. Furthermore, our study did not analyze different viral mutants nor did it account for specific ICU patient management strategies such as ventilation, proning or sedation strategies. These all might have contributed to the lower observed overall mortality in the low anti-Xa cohort. The lower anti-Xa target was deployed later in the COVID-19 pandemic in response to the higher rates of ICH observed in earlier phases of the pandemic. Eligibility criteria for VV ECMO and practices for ICU and ventilator management during VV ECMO support evolved to some degree at the participating centers over the course of the pandemic, potentially contributing to higher survival rates in the lower anti-Xa group.

## Conclusions

A less intense anticoagulation target (anti-Xa activity 0.15–0.3 U/mL) was associated with a decreased incidence of ICH and lower mortality in COVID-19 patients on VV ECMO support. Our results highlight the need for prospective studies to evaluate anticoagulation regimens in ARDS patients on ECMO support.

## Supplementary Information

**Additional file 1: Table S1.** Linear mixed effect model for the prediction of the anti-Xa activity.

**Additional file 2: Table S2.** Competing risk regression model for intracranial hemorrhage treating death without intracranial hemorrhage as a competing event and study site as a frailty term.

## Data Availability

The datasets used and/or analyzed during the current study are available from the corresponding author on reasonable request.

## Abbreviations

anti-Xa: Anti-factor Xa activity
ARDS: Acute respiratory distress syndrome
CCT: Cranial computed tomography
ELSO: Extracorporeal Life Support Organization
ICH: Intracerebral hemorrhage
ICU: Intensive care unit
IL-6: Interleukin 6
RT-PCR: Real-time reverse transcriptase-polymerase chain reaction
SOFA score: Sequential Organ Failure Assessment score
UFH: Unfractionated heparin
VV ECMO: Venovenous extracorporeal membrane oxygenation
