# Supplementary material for: Reduced anticoagulation strategy is associated with a lower incidence of intracerebral hemorrhage in COVID-19 patients on extracorporeal membrane oxygenation
Source: Intensive Care Med Exp. 2023 Jun 12;11:38. doi: 10.1186/s40635-023-00525-3 (PMC10257972; doi:10.1186/s40635-023-00525-3)
Supplement: Supplementary file 1 — Additional file 1: Table S1. Linear mixed effect model for the prediction of the anti-Xa activity. [file 40635_2023_525_MOESM1_ESM.docx]

**Table S1: Linear mixed effect model for the prediction of the Anti-Xa activity**

|  | **Variance** | **Standard Deviation** | |
| --- | --- | --- | --- |
| **Random Effect** | | | |
| ***Intercept* (Patient) nested in center** | 0.04975 | 0.223043 | |
| ***Intercept* Center** | 0.0002615 | 0.016170 | |
|  |  |  | |
|  | **Estimate** | **Standard Error** | **p** |
| **Fixed Effects** | | | |
| ***Intercept*** | 0.61296 | 0.04571 | <0.001 |
| **Lower anticoagulation group (yes)** | -0.30896 | 0.05837 | <0.001 |
| **Time (days)** |  |  |  |
| **Days 1-2** | -0.35728 | 0.07671 | <0.001 |
| **Days 3-7** | -0.04335 | 0.05094 | 0.412 |
| ***Interaction:* Lower AC group – Days 1-2** | 0.30687 | 0.09570 | 0.002 |
| ***Interaction:* Lower AC group – Days 3-7** | 0.01933 | 0.06167 | 0.754 |
