# Supplementary material for: Reduced anticoagulation strategy is associated with a lower incidence of intracerebral hemorrhage in COVID-19 patients on extracorporeal membrane oxygenation
Source: Intensive Care Med Exp. 2023 Jun 12;11:38. doi: 10.1186/s40635-023-00525-3 (PMC10257972; doi:10.1186/s40635-023-00525-3)
Supplement: Supplementary file 2 — Additional file 2: Table S2. Competing risk regression model for intracranial hemorrhage treating death without intracranial hemorrhage as a competing event and study site as a frailty term. [file 40635_2023_525_MOESM2_ESM.docx]

**Table S2: Competing risk regression model for intracranial hemorrhage treating death without intracranial hemorrhage as a competing event and study site as a frailty term.**

|  | **Estimate (Standard error)** | **Subhazard ratio (97.5% CI)** | **p** |
| --- | --- | --- | --- |
| **Variables** | | | |
| **Lower anticoagulation group** | -1.221 (0.61) | 0.295 (0.09-0.97) | 0.044 |
| **SOFA Score (per point)** | 0.029 (0.05) | 1.029 (0.93-1.14) | 0.573 |
| **Female** | -0.301 (0.44) | 0.740 (0.31-1.75) | 0.494 |
| **Dexamethasone** | 0.234 (0.42) | 1.263 (0.56-2.86) | 0.582 |
| **Tocilizumab** | -0.778 (1.11) | 0.459 (0.05-4.07) | 0.387 |

Abbreviations: CI – confidence interval; SOFA: sequential organ failure assessment score
